# Fibroblast Growth Factor 21 Levels Exhibit the Association With Renal Outcomes in Subjects With Type 2 Diabetes Mellitus

**DOI:** 10.3389/fendo.2022.846018

**Published:** 2022-04-21

**Authors:** Li-Hsin Chang, Chia-Huei Chu, Chin-Chou Huang, Liang-Yu Lin

**Affiliations:** ^1^ Division of Endocrinology and Metabolism, Department of Medicine, Yeezen General Hospital, Taoyuan, Taiwan; ^2^ Department of Medical Laboratory Science and Biotechnology, Yuanpei University of Medical Technology, Hsinchu, Taiwan; ^3^ Department of Otorhinolaryngology-Head and Neck Surgery, Mackay Memorial Hospital, Taipei, Taiwan; ^4^ Department of Audiology and Speech Language Pathology, Mackay Medical College, New Taipei City, Taiwan; ^5^ Division of Cardiology, Department of Medicine, Taipei, Taiwan; ^6^ Faculty of Medicine, National Yang Ming Chiao Tung University, Taipei, Taiwan; ^7^ Division of Endocrinology and Metabolism, Department of Medicine, Taipei, Taiwan

**Keywords:** fibroblast growth factor 21, type 2 diabetes mellitus, renal outcomes, biomarker, diabetic kidney disease

## Abstract

**Background:**

Whether microalbuminuria predicts renal outcomes in patients with type 2 diabetes mellitus (T2DM) is argued. Fibroblast growth factor 21 (FGF-21) levels were elevated by the pathogenic process of diabetic kidney disease. The purpose of the study was to evaluate the associations of FGF-21 and renal outcomes in subjects with T2DM.

**Methods:**

Chinese patients with T2DM were enrolled and then observed prospectively, and FGF-21 levels at baseline were measured. The associations of FGF-21 levels and renal composite events, defined by a drop > 30% of eGFR or worsening category of albuminuria, were evaluated using Cox analysis. The appropriate cut-off value of FGF-21 was mapped by the receiver operating characteristic (ROC) curve.

**Results:**

Among 312 subjects, higher FGF-21 levels were associated with higher risks of renal events in Cox analysis. The area under the curve of FGF-21 levels in the ROC curve was optimal (0.67, p < 0.001), and the cut-off value of 1.40 pg/dl exhibited the best sensitivity (76.2%) and specificity (53.5%). The frequency of renal composite events was higher in subjects with FGF-21 ≥ 1.40 pg/dl than in others (30% vs. 10%, p<0.001 by the log-rank test). The worse renal outcomes predicted by FGF-21 ≥ 1.40 pg/dl were confirmed using the adjustments of Cox sequential models (hazard ratio 2.28, 95% confidence interval 1.23–4.24, p=0.009) and consistent across subjects with different status of baseline characteristics and renal risks.

**Conclusion:**

FGF-21 levels were proportional to the risks of renal events in broad- spectrum Chinese T2DM subjects, making it a potential biomarker to predict the renal outcomes of T2DM.

## Introduction

The estimated number of patients with type 2 diabetes mellitus (T2DM) was about 2,300,000 in 2014, and the prevalence increased by 66% from 2005 to 2014 in Taiwan (1). The trend of rising numbers of the subjects with T2DM in Taiwan is consistent with the increasing prevalence of T2DM globally and nearly one-fifth of subjects with T2DM in Taiwan were diagnosed with diabetic kidney disease (DKD) (2, 3). The managements of renal risk of Chinese subjects with T2DM were extremely crucial because the cross-sectional study to evaluate the true prevalence of albuminuria through the sampling of spot urine showed that Asian subjects were more susceptible to DKD than Caucasians. The end-stage renal disease is the tragic ending course of DKD, and the DKD is still the leading etiology of end-stage renal disease worldwide (4, 5). Furthermore, the end-stage renal disease caused by DKD is associated with the loss of life. The UK Prospective Diabetes Study reported that one-fifth of subjects with end-stage renal disease resulting from DKD die annually (6).

Diabetic nephropathy can be detected by the method of sequential tests of the urinary albumin levels, and the rising levels of albuminuria were related to the worse prognosis of the kidney (7, 8). However, the association of the proportional increment of urinary albumin excretion and worse renal outcomes was unreliable when the excretion of urinary albumin was less than 300 mg per day because the fluctuations of urinary albumin levels became obvious. Moreover, the magnitude of reducing microalbuminuria through intensive glycemic or blood pressure control was not related to the mitigation of renal or cardiovascular risk in some cohorts (9, 10). Besides, the decline of the renal functions was observed in a portion of the subjects with T2DM without preceding albuminuria (11). Since the predictions of renal outcomes by the urinary albumin levels within normo- or micro-albuminuria were limited, whether other biomarkers involved in the pathogenesis of DKD can stratify renal risk accurately became a critical issue.

Fibroblast growth factor 21 (FGF-21), produced by hepatocytes and cleared by the kidney, primarily acts as a metabolic regulator to increase insulin sensitivity, cellular glucose uptake, and lipid metabolism (12). However, the extrinsic and intrinsic stressors related to the pathogenesis of DKD, such as hypoxia or oxidative stress on mitochondria, can facilitate the production of FGF-21 (13). Furthermore, the levels of FGF-21 increase in proportion to the progression of the stage of albuminuria in subjects with T2DM (14). Therefore, FGF-21 was a potential biomarker to predict the real outcomes in diabetic cohorts.

Base on the aforementioned message and hypothesis, we tested whether the levels of FGF-21 can predict renal events in the cohort of Chinese subjects with T2DM in this study.

## Material and Methods

### Patient Population and Medical Records

The prospectively observational study focused on the investigation of the correlations of the clinical or biochemical characters and renal outcomes among the Chinese subjects with T2DM was conducted in the Department of Endocrinology and Metabolism of the Taipei Veterans General Hospital since June 2014. We enrolled the subjects who were diagnosed with T2DM and willing to participate in the observational program, but those with chronic kidney disease (CKD) stage IV or above, with acute hepatitis caused by an unknown reason, or with hemoglobin A1c (HbA1c) more than 9% were excluded. The definition of CKD was based on the classification reported from Kidney Disease: Improving Global Outcomes according to the values of the estimated glomerular filtration rate (eGFR) (15). The detailed clinical assessments, including the past history, demographic, and anthropometric markers relevant to the complications of T2DM, were conducted to each subject in the moment of participation. The laboratory tests of traditional markers associated with diabetic complications were collected in the first visit, and the samples of serum creatinine, spot urinary creatinine, and spot urinary albumin were obtained initially from the subjects after fasting for 8–12 h and then collected every 6 months. The human Enzyme-linked immunosorbent assay (ELISA) kits were used to measure the levels of FGF-21 (Cat. No.: RD191108200R, BioVendor, Brno, Czech Republic). The coefficient of variation of the ELISA kit was 3.0%–4.1% for the intra-assay and 3.6%–3.9% for the inter-assay. The FGF-21 can be detected by the kit when the level is above 0.07 pg/dl. The study protocol was approved by the institutional review board of the Taipei Veterans General Hospital, Taiwan.

### Definition of the Renal Composite Events

The renal composite events were defined as the first occurrence of the worsening eGFR or category of albuminuria. The modification of diet in renal disease (MDRD) was used as the method to calculate eGFR (16). Worsening eGFR was recognized as a drop of more than 30% levels of eGFR compared with the baseline and confirmed by a sequential test 6 months later, and this definition was used as one of the components of renal events in the clinical trial (17). The category of albuminuria was defined as normoalbuminuria, microalbuminuria, and macroalbuminuria, and the status of albuminuria was determined by the spot urinary albumin-to-creatinine ratio (UACR) of the baseline. Normoalbuminuria means that the UACR is less than 30 mg/g creatinine, and for microalbuminuria, the UACR is between 30 and 300 mg/g creatinine, as well as macroalbuminuria is defined as the UACR with more than 300 mg/g creatinine (18). The worsening category of albuminuria included progressive shifts from normoalbuminuria to microalbuminuria, microalbuminuria to macroalbuminuria, or normoalbuminuria to macroalbuminuria, and the shifts should be validated by the sequential result of UACR 6 months after the previous report. The definitions of the worsening category of albuminuria in our study were based on one of the renal endpoints in the previous literature (19).

### Statistics

Continuous variables were expressed as mean ± standard deviation (SD), and categorical variables were presented as the numbers and percentages. The analysis of variance was used to compare the values of continuous variables, and Pearson’s chi-squared test was the method utilized to compare the numbers of the categorical variables across the subjects with and without renal composite events. Kaplan–Meier analysis was used to calculate the cumulative the event-free probabilities of renal composite events, and the discrepancies of the survival probability across FGF-21 levels were identified by a log-rank test. The univariate Cox regression analysis was conducted to confirm the associations of variables and the risks of occurrences of renal composite events. The cut-off value of FGF-21 with the best sensitivity and specificity for predicting the occurrences of the renal composite events was identified by a receiver operating characteristic (ROC) curve and the maximum value of Youden’s index, which was defined as sensitivity (1—specificity). Considering that the timelines of events may interfere with the result, the cut-off value was calculated by years for confirming the value generated from that overall cohort can predict the occurrences of the renal composite events year by year. Three Cox models were constructed sequentially for confirming the associations of the levels of FGF-21 and the risks of renal composite events. The associations of FGF-21 levels and the risks of renal composite events were adjusted by age and gender in model 1 and adjusted by age, gender, and the duration of diabetes in model 2. In model 3, the associations were adjusted by all factors relevant to the renal prognosis of this study. If the associations between FGF-21 levels and renal composite events were proven, further univariate Cox regression analyses and sequential Cox models were conducted for testing the associations between FGF-21 levels and worsening albuminuria or declines of eGFR. All aforementioned data were analyzed with IBM SPSS software 18.0.

## Results

### Baseline Characteristics

**Figure 1:**
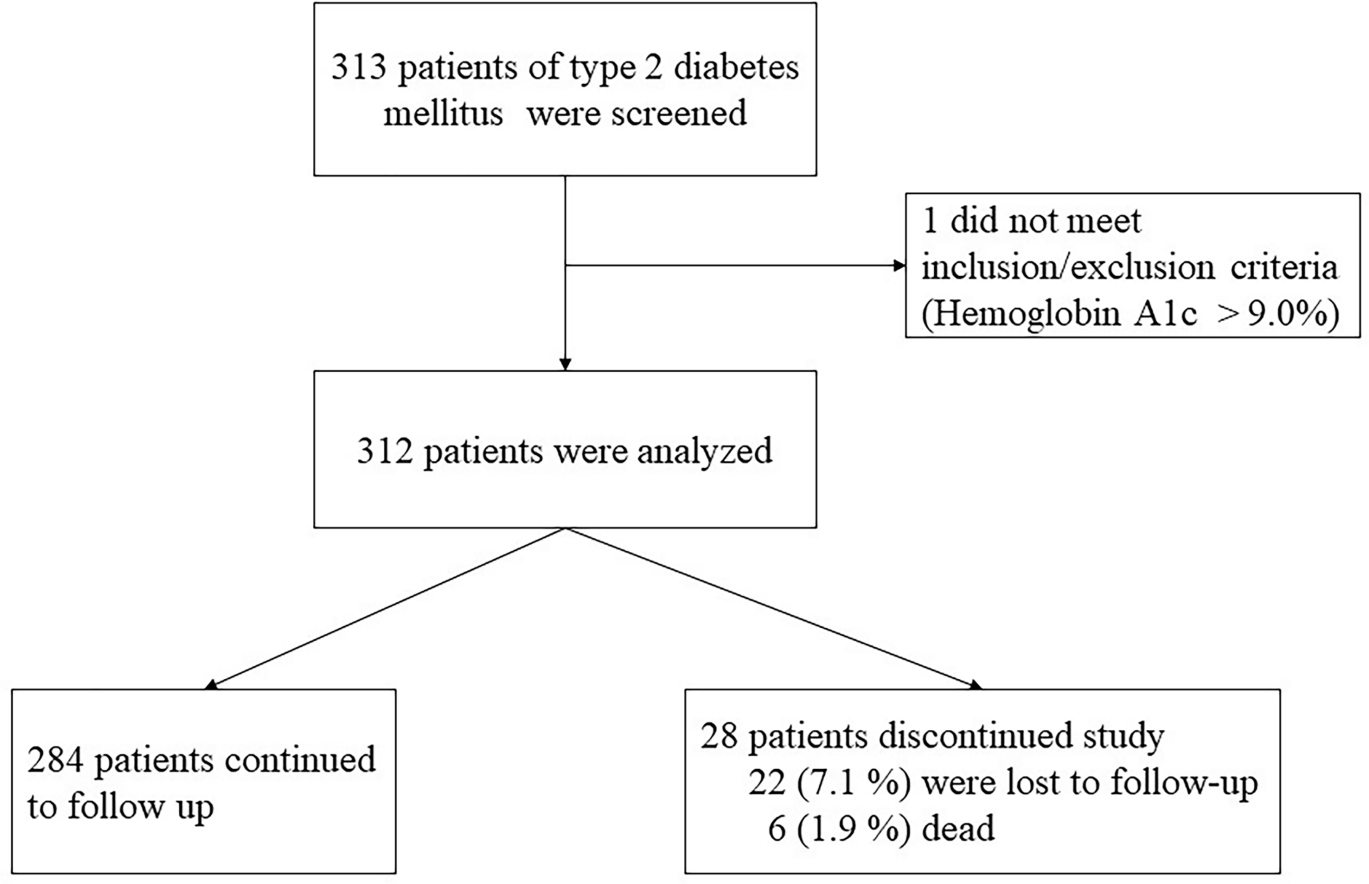
Patient disposition.

**Figure 2:**
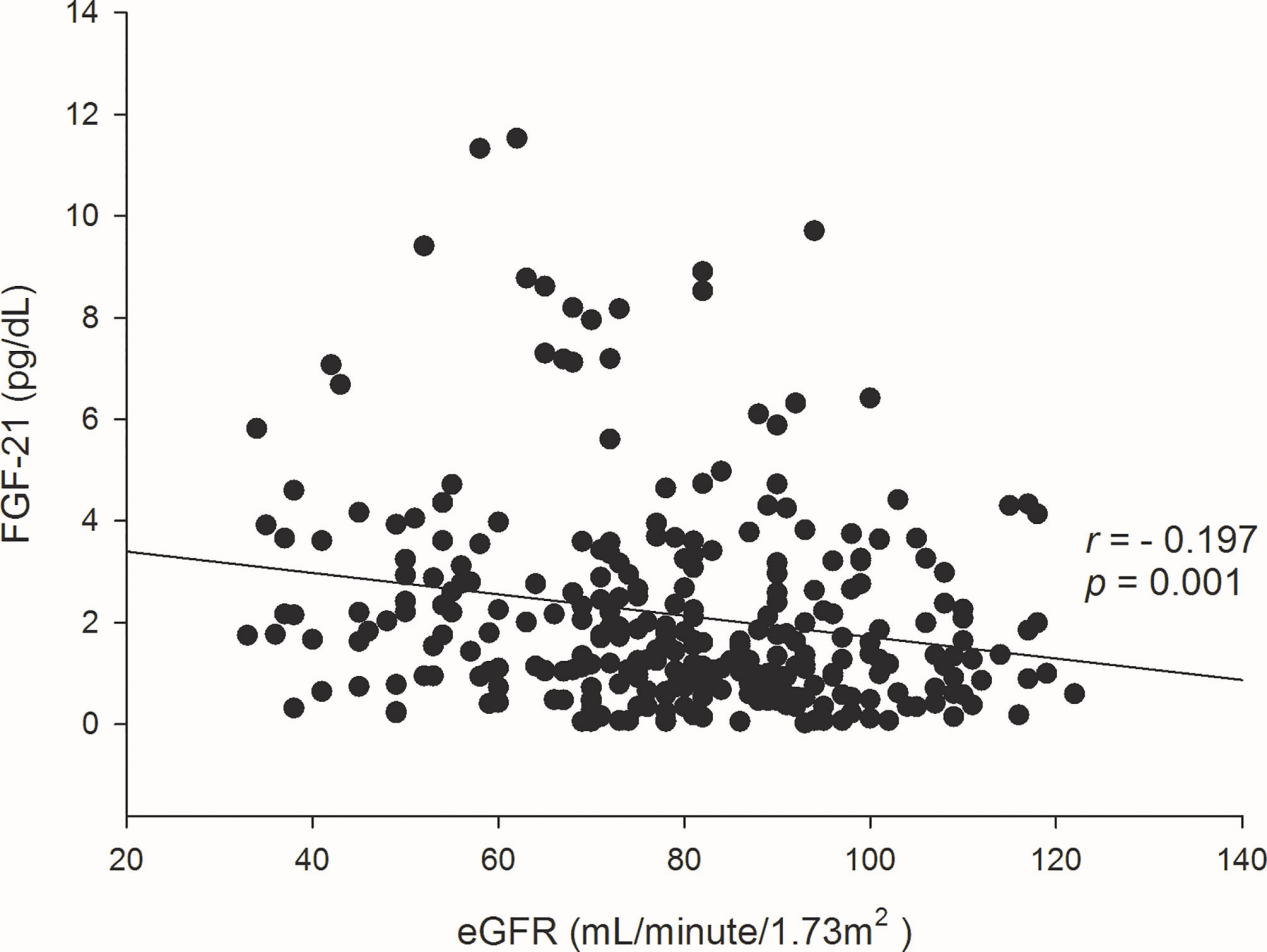
Scatter plots and correlation efficient (*r*) of the concentrations of FGF-21 and eGFR levels at baseline.

Three hundred thirteen subjects with T2DM were recruited in this study, and the last patient analyzed in this study participated in the program on April 3, 2019. One subject whose HbA1c is more than 9% was excluded (**Figure 1**). Among 312 subjects with a mean age of 61.2 years old, hypertension and hyperlipidemia, which accounted for 60% and 80% of subjects in this cohort, were the most common comorbidities and one out of ten had retinopathy or neuropathy. Nearly one-third subjects in this cohort were at moderate to high renal risk. The prevalence of albuminuria was 33%, CKD stage 3 or above was 20%, and 10% of subjects had both CKD stage 3 or above and albuminuria. The management of glycemia and blood pressure was optimal; the mean HbA1c was 7.0%, and the mean blood pressure was 114 mmHg. Metformin and sulfonylurea were most commonly prescribed oral anti-hyperglycemic agents in our cohort of patients, and 17% of the subjects were treated by subcutaneous insulin injection. For other medications that were relevant to the renal outcomes, nearly half of the subjects received renin–angiotensin system (RAS) inhibitors and only 3 subjects received the therapy of inhibitors of sodium-glucose co-transporter type 2 (SGLT2i) (**Table 1**). No one was treated by the analogues of glucagon-like peptide-1 (GLP1a). The concentrations of FGF-21 were negatively correlated with the eGFR levels (correlation efficient -0.197, *p* =0.001) (**Figure 2**). The mean value and standard deviation of FGF-21 level were 1.91 ± 1.73 pg/dl for the first tertile of eGFR group (eGFR 90-120 ml/min/1.73m^2^), 1.91 ± 1.80 pg/dl for the second tertile of eGFR group (eGFR 72-89 ml/min/1.73m^2^), and 2.74 ± 2.54 pg/dl for the third tertile of eGFR group (eGFR 33-71 ml/min/1.73m^2^). The FGF-21 levels were significantly higher in the third tertile group of eGFR (*p*=0.005 according to the analysis of variance).

**Table 1:** Baseline characteristics of subjects with T2DM grouped by the occurrence of renal composite events or not.

	All (312)	Renal composite events		*p*-value
		Yes (n = 66)	No (n = 246)	
Age	61.2 ± 13.0	65.3 ± 11.1	60.0 ± 13.2	0.003
Male sex (%)	213 (68)	38 (57)	175 (71)	0.03
Smoking (%)	94 (30)	16 (24)	78 (31)	0.40
Coronary artery disease (%)	60 (19)	18 (27)	42 (17)	0.07
Hyperlipidemia (%)	267 (85)	55 (83)	212 (86)	0.55
Hypertension (%)	188 (60)	48 (72)	140 (57)	0.02
Retinopathy (%)	30 (9)	14 (21)	16 (6)	0.001
Neuropathy (%)	32 (10)	11 (16)	21 (8)	0.06
Albuminuria (%)	103 (33)	35 (53)	68 (27)	<0.001
CKD stage 3 (%)	64 (20)	24 (36)	40 (16)	0.001
CKD stage 3 with albuminuria (%)	36 (11)	17 (25)	19 (7)	<0.001
Duration of diabetes (years)	11.2 ± 7.7	14.2 ± 9.4	10.4 ± 6.9	<0.001
Body mass index	26.2 ± 4.5	25.8 ± 4.3	26.3 ± 4.5	0.44
Waist-to-hip ratio	0.93 ± 0.06	0.93 ± 0.07	0.93 ± 0.06	0.60
Systolic blood pressure (mmHg)	132 ± 15	134 ± 15	132 ± 15	0.31
Diastolic blood pressure (mmHg)	78 ± 11	78 ± 11	78 ± 11	0.93
Mean blood pressure (mmHg)	114 ± 12	116 ± 12	114 ± 13	0.43
Urinary albumin-to-creatinine ratio (mg/g Cr)	111.4 ± 321.2	266.9 ± 540.4	70.3 ± 214.7	<0.001
HbA1c (%)	7.0 ± 0.8	7.1 ± 0.8	7.0 ± 0.8	0.44
Total cholesterol (mg/dl)	168 ± 27	167 ± 24	168 ± 27	0.75
Creatinine (mg/dl)	0.95 ± 0.26	1.02 ± 0.34	0.93 ± 0.24	0.02
eGFR (ml/min/1.73m^2^)	78 ± 19	69 ± 19	80 ± 19	<0.001
FGF-21 (pg/dl)	2.17 ± 2.07	3.32 ± 2.81	1.87 ± 1.72	<0.001
Metformin (%)	239 (76)	48 (72)	191 (77)	0.41
Sulfonylurea (%)	121 (38)	27 (41)	94 (38)	0.77
Dipeptidyl peptidase-4 inhibitor (%)	54 (17)	15 (22)	39 (16)	0.20
SGLT2 inhibitor (%)	3 (0.9)	1 (1)	2 (0.8)	0.51
Insulin (%)	55 (17)	14 (21)	41 (16)	0.37
Renin-angiotensin system blockade (%)	154 (49)	45 (68)	109 (44)	0.001
Diuretics (%)	39 (12)	15 (22)	24 (9)	0.01
eGFR decline > 30% (%)		22 (33)		
Worsening albuminuria (%)		44 (66)		
Normo- to microalbuminuria (%)		21 (32)		
Micro- to macroalbuminuria (%)		22 (33)		
Normo- to macroalbuminuria (%)		1 (2)		

Data are expressed as numbers and percentage in non-continuous variables and mean ± SD in continuous variables,

p < 0.05 was defined as significant difference among two groups.

CKD, chronic kidney disease; FGF-21, fibroblast growth factor 21; SGLT2, sodium glucose cotransporter type 2; HbA1c, hemoglobin A1C; eGFR, estimated glomerular filtration rate.

### FGF-21 and the Renal Composite Events of Type 2 Diabetes

**Figure 3:**
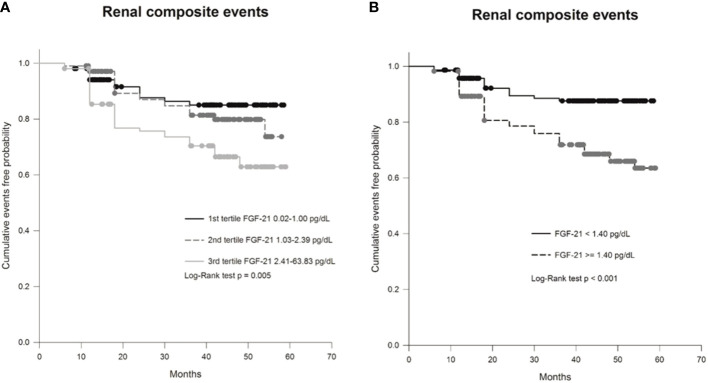
Kaplan-Meier curve showing the probability of cumulative event-free survival of renal events (either or both a decline of >30% in eGFR and worsening stage of albuminuria) in **(A)** patients with type 2 diabetes (T2DM) divided into groups based on concentration tertiles of FGF-21 levels and in **(B)** patients with T2DM grouped based on a specific cut-off value of FGF-21 (≥1.40 pg/dL vs. < 1.40 pg/dL).

Subjects were followed for a median of 3.5 years, and their vital status was analyzed on October 2019. Twenty-two subjects were lost to follow-up, and 5 subjects died because of malignancy as well as 1 individual died due to the infection of the respiratory tract. Sixty-six renal composite events occurred; in 22 events, there was a decline of more than 30% of eGFR, and 44 events were the worsening category of albuminuria. Those with the occurrences of the renal composite events had unfavorable conditions, such as being an elder, a greater prevalence of vascular complications of diabetes, higher UACRs, and lower eGFR at baseline, compared with individuals free from the renal composite events. However, the uses of RAS blockade for individuals with occurrences of the renal composite events were more extensive (**Table 1**). The tertile group with the highest concentration of FGF-21 had the highest risk of renal composite events (p < 0.001 by log-rank test; **Figure 3A**). In univariate Cox analysis, FGF-21 levels were significantly associated with the risks of the renal composite events (HR 1.22, 95% CI 1.12–1.33, *p <* 0.001). The factors of age, gender, the duration of diabetes, levels of UACR and eGFR, history of retinopathy, and uses of RAS blockers or diuretics, were also relevant to the risk of occurrences of the renal composite events in this cohort. FGF-21 levels were still associated with the rate of the renal composite events after adjusting aforementioned relevant factors in Cox sequential models (HR 1.25, 95% CI 1.12–1.39, p < 0.001). Besides, FGF-21 levels predicted the occurrences of worsening category of albuminuria and the results were verified by univariate Cox analysis and Cox sequential models (unadjusted HR 1.27, 95% C.I. 1.15–1.40, p < 0.001; adjusted HR 1.23, 95% C.I 1.06–1.42, p = 0.006). The trends of increasing levels of FGF-21 and risks of declines of renal function were consistent to the associations between FGF-21 levels and risks of renal composite events but did not reach the statistical significance (unadjusted HR 1.05, 95% C.I. 0.87–1.27, p = 0.58) (**Table 2**).

**Table 2:** Result of univariate and multivariate Cox proportional hazard model for association of FGF-21 with renal composite events.

Renal composite events	HR	95% CI	*p-*value
Univariate analysis			
FGF-21(pg/dl)	1.22	1.12–1.33	<0.001
FGF-21 ≥ 1.40 pg/dl	2.78	1.55–4.96	0.001
Age	1.02	1.00–1.04	0.03
Male sex	1.68	1.03–2.74	0.03
Duration of diabetes (years)	1.04	1.01–1.07	0.003
Retinopathy (with versus without)	2.55	1.41–4.60	0.002
eGFR (ml/min/1.73m^2^)	0.97	0.96–0.99	0.001
UACR (per 100 mg/g Cr increase) (mg/g Cr)	1.09	1.04–1.14	<0.001
RAS inhibitors (with versus without)	2.19	1.30–3.68	0.003
Diuretics (with versus without)	2.13	1.20–3.79	0.01
**Multivariate analysis for FGF-21 (pg/dl)**			
**Model 1**			
FGF-21 (pg/dl)	1.21	1.11–1.32	<0.001
**Model 2**			
FGF-21 (pg/dl)	1.21	1.10–1.34	<0.001
Duration of diabetes (years)	1.04	1.01–1.07	0.002
**Model 3**			
FGF-21 (pg/dl)	1.25	1.12–1.39	<0.001
Duration of diabetes (years)	1.03	1.00–1.07	0.01
UACR (per 100 mg/g Cr increase) (mg/g Cr)	1.06	1.01–1.12	0.01
**Multivariate analysis for FGF-21 ≥ 1.40 pg/dl**			
**Model 1**			
FGF-21 ≥ 1.40 pg/dl	2.78	1.55–4.96	0.001
**Model 2**			
FGF-21 ≥ 1.40 pg/dl	2.77	1.52–5.04	0.001
Duration of diabetes (years)	1.03	1.01–1.06	0.007
**Model 3**			
FGF-21 ≥ 1.40 pg/dl	2.28	1.23–4.24	0.009
Duration of diabetes (years)	1.03	1.00–1.06	0.04
UACR(per 100 mg/g Cr increase) (mg/g Cr)	1.05	1.00–1.10	0.02
**Worsening albuminuria**			
**Univariate analysis for** **FGF-21 and FGF-21 ≥ 1.40 pg/dl**			
FGF-21(pg/dl)	1.27	1.15–1.40	<0.001
FGF-21 ≥ 1.40 pg/dl	3.08	1.48–6.42	0.003
**Multivariate analysis for FGF-21 (pg/dl)**			
**Model 1**			
FGF-21 (pg/dl)	1.25	1.13–1.38	<0.001
**Model 2**			
FGF-21 (pg/dl)	1.25	1.11–1.40	<0.001
**Model 3**			
FGF-21 (pg/dl)	1.23	1.06–1.42	0.006
**Multivariate analysis for FGF-21 ≥ 1.40 pg/dl**			
**Model 1**			
FGF-21 ≥ 1.40 pg/dl	2.73	1.30–5.73	0.008
**Model 2**			
FGF-21 ≥ 1.40 pg/dl	2.58	1.22–5.44	0.01
Duration of diabetes (years)	1.05	1.00–1.10	0.02
**Model 3**			
FGF-21 ≥ 1.40 pg/dl	2.07	0.94–4.57	0.70
**>30% decline in eGFR**			
**Univariate analysis for** **FGF-21 and FGF-21 ≥ 1.40 pg/dl**			
FGF-21 (pg/dl)	1.05	0.87–1.27	0.58
FGF-21 ≥ 1.40 pg/dl	2.04	0.80–5.23	0.13

Model 1: adjusted for age and sex.

Model 2: adjusted for age, sex, and duration of diabetes.

Model 3: adjusted for age, sex, duration of diabetes, retinopathy, eGFR, UACR, treatment with RAS inhibitors, and treatment with diuretics.

FGF-21, fibroblast growth factor 21; HR, hazard ratio; CI, confidence interval; eGFR, estimated glomerular filtration rate; UACR, urinary albumin-to-creatinine ratio; Cr, creatinine; RAS, renin–angiotensin system.

**Figure 4:**
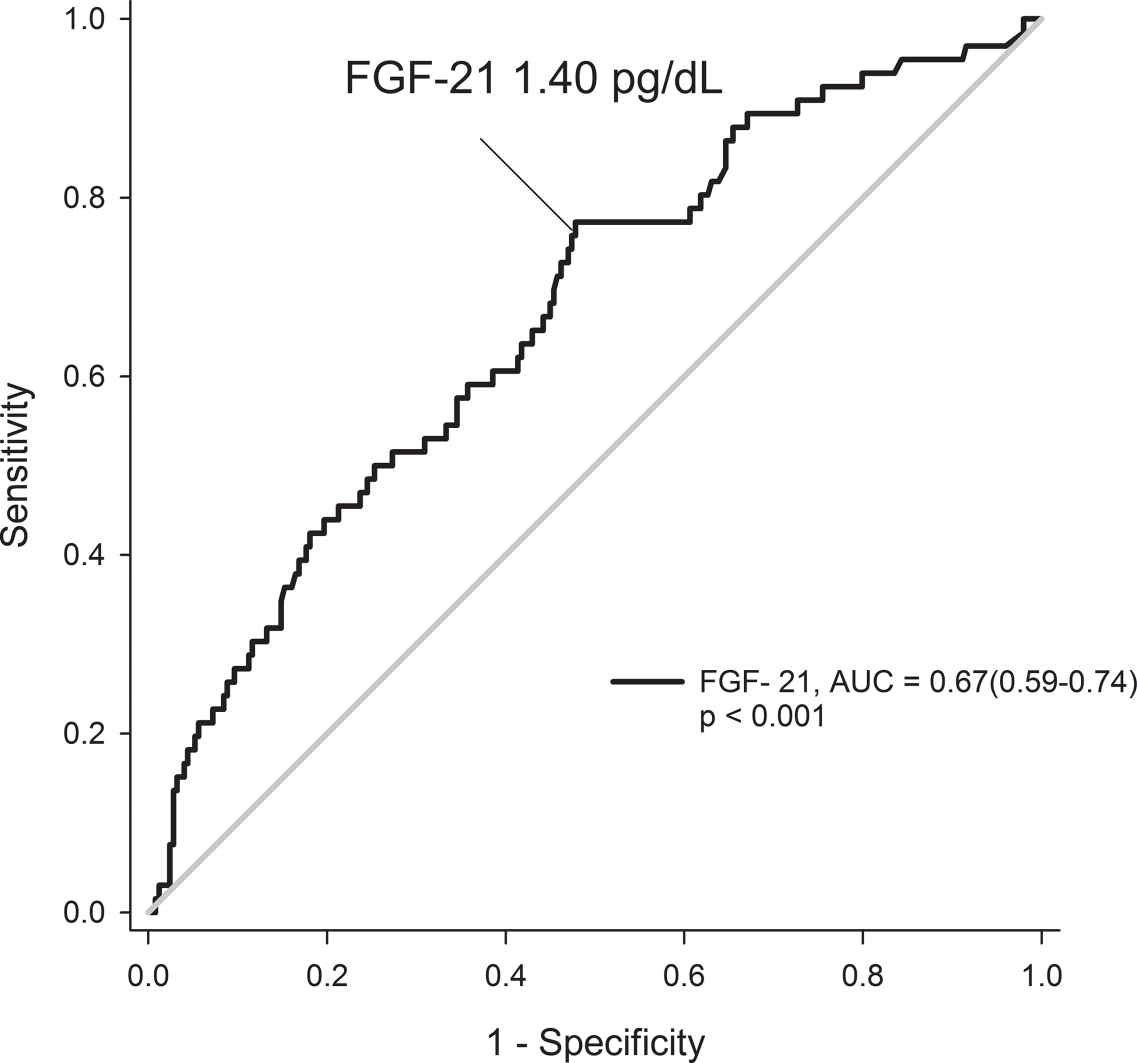
Receiver operating characteristic curves for prediction of renal events by FGF-21 levels in patients with type 2 diabetes.

The ROC curve of FGF-21 levels further proved the levels of FGF-21 can predict occurrences of the renal composite events by exhibiting area under curve 0.67 which showed statistical significance (95% CI 0.59–0.74, p < 0.001; **Figure 4**). Furthermore, the ROC curve mapping out the specific values of FGF-21 with maximal sensitivity and specificity and demonstrated a 1.40 pg/dl generated a sensitivity of 76.2% and a specificity of 53.3%. The areas under curve showed consistent results from the first to the fifth year. The maximum values of Youden’s index were 0.28-0.30 and the best cut-off value of FGF-21 was a 3.42 pg/dl for the first year then showed a 1.40 pg/dl from the second year till the final date of follow-up in our study (**Supplementary Figure 1**).

### FGF-21 ≥ 1.40 pg/dl and Renal Composite Events

Subjects with FGF-21 levels of ≥1.40 pg/dl had greater portion of hypertension, albuminuria, or advanced CKD but the uses of RAS blockade were also more common comparing with those with FGF-21 < 1.40 pg/dl (56% vs 41%, p = 0.009) (**Table 3**). Individuals with FGF-21 ≥ 1.40 pg/dl had higher rates of renal composite events occurring than those with FGF-21 < 1.40 pg/dl (30% vs. 10%, p < 0.001 by log-rank test; **Figure 3B**). The trends toward the increased risks of a decline of more than 30% of eGFR or progressively shifting category of albuminuria in subjects with FGF-21 ≥ 1.40 pg/dl were consistent with the primary endpoint of renal composite events (**Table 3**).

**Table 3:** Baseline characteristics and renal outcomes of subjects with T2DM grouped by FGF-21.

	All (312)	FGF-21		*p*-value
		<1.40 pg/dl (n = 143)	≥1.40 pg/dl (n = 169)	
Age	61.2 ± 13.0	59.7 ± 12.8	62.4 ± 13.0	0.07
Male gender (%)	213 (68)	106 (74)	107 (63)	0.05
Smoking (%)	94 (30)	49 (34)	45 (26)	0.27
Coronary artery disease (%)	60 (19)	24 (16)	36 (21)	0.38
Hyperlipidemia (%)	267 (85)	124 (86)	143 (84)	0.63
Hypertension (%)	188 (60)	80 (56)	108 (64)	0.002
Retinopathy (%)	30 (9)	9 (6)	21 (12)	0.08
Neuropathy (%)	32 (10)	14 (10)	18 (10)	0.85
Albuminuria (%)	103 (33)	38 (26)	65 (38)	0.02
CKD stage 3 (%)	64 (20)	14 (10)	50 (29)	<0.001
CKD stage 3 with albuminuria (%)	36 (11)	8 (5)	28 (16)	0.002
Duration of diabetes (years)	11.2 ± 7.7	10.3 ± 7.7	11.9 ± 7.5	0.07
Body mass index	26.2 ± 4.5	26.2 ± 4.5	26.1 ± 4.5	0.86
Waist-to-hip ratio	0.93 ± 0.06	0.93 ± 0.07	0.93 ± 0.06	0.93
Systolic blood pressure (mmHg)	132 ± 15	131 ± 14	133 ± 16	0.23
Diastolic blood pressure (mmHg)	78 ± 11	78 ± 9	79 ± 12	0.31
Mean blood pressure (mmHg)	114 ± 12	113 ± 11	115 ± 13	0.21
Urinary albumin-to-creatinine ratio (mg/g Cr)	111.4 ± 321.2	77.2 ± 217.6	141.2 ± 387.9	0.08
HbA1c (%)	7.0 ± 0.8	7.0 ± 0.8	7.0 ± 0.8	0.94
Total cholesterol (mg/dl)	168 ± 27	163 ± 26	172 ± 28	0.008
Creatinine (mg/dl)	0.95 ± 0.26	0.90 ± 0.20	1.00 ± 0.30	0.001
eGFR (ml/min/1.73m^2^)	78 ± 19	83 ± 16	74 ± 20	<0.001
FGF-21 (pg/dl)	2.85 ± 5.04	0.71 ± 0.39	4.66 ± 6.30	NA
Metformin (%)	239 (76)	114 (79)	125 (74)	0.28
Sulfonylurea (%)	121 (38)	50 (35)	71 (42)	0.24
Dipeptidyl peptidase-4 inhibitor (%)	54 (17)	20 (14)	34 (20)	0.17
SGLT2 inhibitor (%)	3 (0.9)	2 (1)	1 (0.5)	0.59
Insulin (%)	55 (17)	24 (16)	31 (18)	0.76
Renin–angiotensin system blockade (%)	154 (49)	59 (41)	95 (56)	0.009
Diuretics (%)	39 (12)	9 (6)	30 (17)	0.003
Renal composite events (%)	66 (21)	15 (10)	51 (30)	<0.001
eGFR decline > 30% (%)	22 (33)	6 (4)	16 (9)	0.07
Worsening albuminuria (%)	44 (66)	9 (6)	35 (21)	<0.001
Normo- to microalbuminuria (%)	21 (32)	6 (4)	15 (9)	0.11
Micro- to macroalbuminuria (%)	22 (33)	3 (2)	19 (11)	0.002
Normo- to macroalbuminuria (%)	1 (2)	0	1 (0.5)	NA

Data are expressed as numbers and percentage in non-continuous variables and mean ± SD in continuous variables,

p < 0.05 was defined as significant difference among two groups.

CKD, chronic kidney disease; FGF-21, fibroblast growth factor 21; HbA1c, hemoglobin A1c; eGFR, estimated glomerular filtration rate; NA, not applicable.

The levels ≥1.40 pg/dl of FGF-21 were associated with the increasing risks of renal composite events (HR 2.78, 95% CI 1.55–4.96, p = 0.001) in Cox analysis. After adjusting the Cox sequential models, the association remained significantly (model 1: HR 2.78, 95% CI 1.55–4.96, p = 0.001; model 2: HR 2.77, 95% CI 1.52–5.04, p = 0.001; model 3: HR 2.28, 95% CI 1.23–4.24, p = 0.009). Furthermore, FGF-2 ≥ 1.40 pg/dl predicted the risks of the worsening category of albuminuria in univariate Cox analysis (HR 3.08, 95% CI 1.48–6.42, p = 0.003) and the trend was consistent, but the significance did not remain after adjusting other variates in sequential Cox models (HR 2.07, 95% CI 0.94–4.57, p = 0.70 for model 3). The FGF-2 ≥ 1.40 pg/dl was also related to the risks of decline of eGFR but did not reach statistical significance (**Table 2**).

The predictions of renal outcomes by levels of FGF-21 were consistent among age being younger or older than 60 years, gender of female or male, systolic blood pressure ≥ or < 140 mmHg, eGFR level higher or lower than 60 ml/minute/1.73m^2^, the level of UACR more or less than 30 mg/g creatinine, and the use of RAS blockers or not (**Table 4**). For 176 subjects with eGFR ≥ 60 ml/min/1.73m^2^ and normoalbuminuria, FGF-21 ≥ 1.40 pg/dl had a tendency to be associated with the increasing risks of renal composite events but did not show the statistical significance in Cox analysis (HR 1.80, 95% CI 0.78–4.12, p = 0.16).

**Table 4:** Results of association of FGF-21 ≧ 1.40 pg/dl and renal composite events stratified by age, sex, blood pressure, eGFR, UACR, and the use of RAS inhibitors.

Renal composite events Subgroup	Crude HR	95% CI	P-value for Interaction
Age ≧ 60 y/o(n=190)	2.18	1.15–4.11	0.88
Age < 60 y/o (n=122)	5.97	1.33–26.69	
Male (n=213)	3.43	1.55–7.55	0.57
Female (n=99)	1.75	0.74–4.13	
SBP ≧ 140 mmHg (n=96)	1.58	0.63–3.96	0.32
SBP < 140 mmHg (n=216)	3.80	1.76–8.19	
eGFR ≧ 60 ml/min/1.73m^2^ (n=247)	2.18	1.15–4.11	0.19
eGFR < 60 ml/min/1.73m^2^ (n = 65)	5.97	1.33–26.69	
UACR ≧ 30 mg/g Cr (n=112)	4.02	1.55–10.41	0.02
UACR < 30 mg/g Cr (n=200)	1.80	0.83–3.87	
RAS inhibitors (n=154)	2.76	1.28–5.97	0.08
No RAS inhibitors (n=158)	2.11	0.84–5.30	

eGFR, estimated glomerular filtration rate; UACR, urinary albumin-to-creatinine ratio; RAS, renin–angiotensin system; SBP, systolic blood pressure; HR, hazard ratio; CI, confidence interval.

## Discussion

Our observational cohort composed of Chinese subjects with T2DM demonstrated that the FGF-21 levels were related to risks of renal composite events defined as the first occurrences of a deterioration of more than 30% in eGFR or the progressively shifting category of albuminuria. Furthermore, the specific cut-off level of FGF-21 (higher than 1.40 pg/dl) also exhibited excellent predictions of the renal outcomes and this association was similar with primary cohort across all major subgroups stratified by the factors that were biologically or statistically relevant to the risks of renal composite events, including age, gender, blood pressure, the status of albuminuria or CKD, and use of blockades of RAS or not.

The associations of FGF-21 levels and renal outcomes in our study were consistent with the previous reports from the United Kingdom and Hong Kong. Looker et al. demonstrated that FGF-21 is one of the 14 biomarkers in addition to the traditional factors, such as age, HbA1C, eGFR, or albuminuria at baseline, that improve the prediction of rapid CKD progression by the design of the case–control study in subjects with T2DM as well as advanced CKD (20). Besides, the reports from Hong Kong, enrolling an equivalent number of subjects with albuminuria and normoalbuminuria, showed that the levels of FGF-21 predict the decline of eGFR in Chinese subjects with T2DM (21). Our study, investigating the subjects with preserved eGFR and fewer portions of albuminuria, further validated that the levels of FGF-21 were relevant to the renal outcomes by a prospectively observational cohort composited by Chinese subjects with T2DM and relatively lower risk of the progression of DKD. Furthermore, the composites of the renal outcomes in our study, which include the deterioration of eGFR and progressive shift of the category of albuminuria, was more consistent to the standard practice that recognized the prevention of both the advancement of CKD and deterioration of albuminuria as crucial targets of diabetic care rather than focusing on the decline of eGFR only. Even if the association between microalbuminuria per se and end-stage renal disease is inconsistent, the shifting stage from normoalbuminuria to microalbuminuria is still recognized as a sign of increasing renal risk such as further progression to macroalbuminuria, which is a validated risk factor of end-stage renal disease (10, 15, 22). The subjects enrolled in our study were mostly free from the status of albuminuria at baseline. Therefore, the stage shifting from normoalbuminuria to microalbuminuria could be interpreted as increasing the risks of further renal damage and was a meaningful sign for intensifying the strategy of preventing complications. Briefly speaking, the associations between the levels of FGF-21 and the renal outcomes in our study suggested that FGF-21 can be a potential marker used in clinical practice to predict the progression of DKD in subjects with T2DM. Anuwatmatee et al. proclaimed that FGF-21 levels were not associated with renal outcomes in a population-based study (23). However, the primary goal of the study was to investigate the associations between the characteristics or biomarkers of the participants and the occurrences of cardiovascular events rather than renal outcomes in subjects free from documented cardiovascular disease at baseline, and only 8%–17% of the subjects had a history of T2DM. Furthermore, the differences of the populations, designs, and definitions of renal events may explain the discrepant results between studies. Our study demonstrated the associations between FGF-21 levels and renal outcomes in the Chinese subjects with T2DM, but whether the conclusions could be generated to non-diabetic subjects recruited from multiple ethnics was unknown. FGF-21 is a renal excreted protein; therefore, the levels of FGF-21 were increased with the decline of eGFR (21). Nevertheless, the constant associations between FGF-21 and renal composite events after adjusting the factors of eGFR levels and consistent results of the subjects with eGFR levels more or less than 60 ml/min/1.73 m^2^ suggested that the predictability of the FGF-21 level was independent rather than provided by the renal function at baseline.

Since there was no distinctly normal range of FGF-21 for subjects with T2DM, a validated cut-off value of FGF-21 that can predict the renal outcomes accurately may facilitate the clinical applications. The levels of FGF-21 more than 1.40 ng/dl demonstrated excellent predictability for the occurrences of the renal events in our cohort before and after adjusting other conventional risk factors in sequential models. Multiple risk factors have been established for predicting the progression of DKD, such as a history of smoking; the urinary excretion of albumin; eGFR at baseline; and glycemic, blood pressure, and lipid level control (24). However, the glycemic and blood pressure levels were not associated with the renal outcomes in our cohort. The explanation of the discrepancy result was possibly due to the balanced control of the plasma glucose and blood pressure in the subjects with and without occurrences of the renal events. Besides, the management of the plasma glucose and blood pressure was relatively optimal in our cohort. The average HbA1c on 7% and mean systolic blood pressure on 135 mmHg were fit to the target according to the suggestions from the clinical guidelines for elder subjects with T2DM (25). The urinary excretion of albumin and the renal function at baseline were strong predictors in our cohort, but the obvious association of the levels of FGF-21 more than 1.40 pg/dl and the renal composite events after adjusting the factors of UACR and eGFR at baseline concluded that the cut-off value of FGF-21 can be a predictor of the renal events without support from the factor of eGFR or UACR. The uses of RAS blockades were related the renal outcomes in our study. The RAS blockade is an essential medicine for preventing the deterioration of DKD in subjects with a high risk of the progression of DKD (26). The higher prevalence of advanced CKD and albuminuria among the subjects with FGF-21 ≥1.40 pg/dl suggested that they were at a high risk of the progression of DKD, and the more extensive coverage of RAS blockade suggested that they were at a more intensive therapy for preventing the deterioration of renal functions. The persistent predictability of the renal events by FGF-21 more than 1.40 pg/dl in the model 3 of regression and consistent associations with FGF-21 ≥1.40 pg/dl and the renal outcomes of the subjects with and without the use of RAS blockades proved that 1.40 pg/dl of FGF-21 can be a threshold to determine whether the subjects with an intensive management of the DKD were at a risk of the occurrence of the renal events or not.

Even the associations between the levels of FGF-21 and the renal outcomes were proven by univariate Cox regression analyses and sequential Cox models; the AUC of the ROC curve was merely reach the criteria of significance advocated the levels of FGF-21 was not the only component to determine the renal risk in our cohort. The result was not surprising and further supported the complicated pathogenesis of DKD. The cascades of the detrimental process after the exposure to hyperglycemia, including the change of tubuloglomerular feedback, hypoxia of renal cells, intra-cellular lipotoxicity, podocytopathy, inflammatory and fibrotic processes, impaired function of mitochondria, overwhelming function of the sodium–hydrogen exchanger, and loss of autophagy, all participated in the genesis and progression of DKD (27–29). The level of FGF-21 was one of surrogate markers that are parallel to the severity of hypoxia, inflammation, and oxidative stress on mitochondria but was not the validated method to estimate the hemodynamic change of the glomerulus or channelopathies of podocytes or renal tubular cells. Therefore, combining other validated markers that are relevant to the pathogenesis of DKD with FGF-21 may exhibit better predictability than FGF-21 alone.

Some weaknesses of the study should be mentioned. First, causal relationships cannot be concluded and there were always unmeasurable confounders from prospectively observational studies. Additional studies are warranted to investigate the role of the FGF-21 pathogenesis of worsening DKD before the occurrences of albuminuria or deteriorations of eGFR. Second, the adherence to crucial medications, such as RAS blockades or diuretics, was not available due to the design of the observational study. Third, the numbers of subjects receiving SGLT2i or GLP-1a were few because the cost of SGLT2i was an out-of-pocket expense until 2016 and the high spending of GLP-1a strangulated the clinical application in Taiwan, even though randomized control studies showed that SGLT2i and GLP1a provide renal protection in subjects with T2DM (30, 31). The effects of the above medications can be clarified by extending the follow-up period in this cohort. Finally, the predictability of biomarkers other than FGF-21 was not tested in our cohort and further investigation should be conducted for confirming the clinical innovations of FGF-21.

## Conclusion

Circulating FGF-21 was associated with renal composite events in a broad-spectrum of Chinese patients with T2DM. Therefore, FGF-21 levels could potentially predict the renal outcomes of subjects with T2DM.

## Data Availability Statement

The raw data supporting the conclusions of this article will be made available by the authors, without undue reservation.

## Ethics Statement

The studies involving human participants were reviewed and approved by the institution review board of the Taipei Veterans General Hospital, Taiwan. The patients/participants provided their written informed consent to participate in this study.

## Author Contributions

L-HC and L-YL were the main conductors of this study and contributed to the study conception and design, implementation, statistical analysis, interpretation, and preparation and finalization of the manuscript. C-HC contributed to the study conception and design, implementation, statistical analysis, and preparation of the manuscript. C-CH contributed to the study conception and design and data collection. All authors approved the final manuscript for publication.

## Funding

This work was supported by research grants V104E11-004-MY2, V105C-131, V107C-201, V108C-197, V109C-179, V110C-198 and V111D62-002-MY3-1 provided to L-YL from Taipei Veterans General Hospital, Taipei, Taiwan, and by research grant No. 2021001 to L-HC from Yeezen General Hospital, Taoyuan, Taiwan.

## Conflict of Interest

The authors declare that the research was conducted in the absence of any commercial or financial relationships that could be construed as a potential conflict of interest.

## Publisher’s Note

All claims expressed in this article are solely those of the authors and do not necessarily represent those of their affiliated organizations, or those of the publisher, the editors and the reviewers. Any product that may be evaluated in this article, or claim that may be made by its manufacturer, is not guaranteed or endorsed by the publisher.

## Supplementary Material

Supplementary Figure 1 Time-dependent receiver operating characteristic curves for prediction of renal events by FGF-21 levels in patients with type 2 diabetes from the first to fifth year **(A–E)** of follow-up.
