# Supplementary figures and images for: Fibroblast Growth Factor 21 Levels Exhibit the Association With Renal Outcomes in Subjects With Type 2 Diabetes Mellitus
Source: Front Endocrinol (Lausanne). 2022 Apr 21;13:846018. doi: 10.3389/fendo.2022.846018 (PMC9069677; doi:10.3389/fendo.2022.846018)

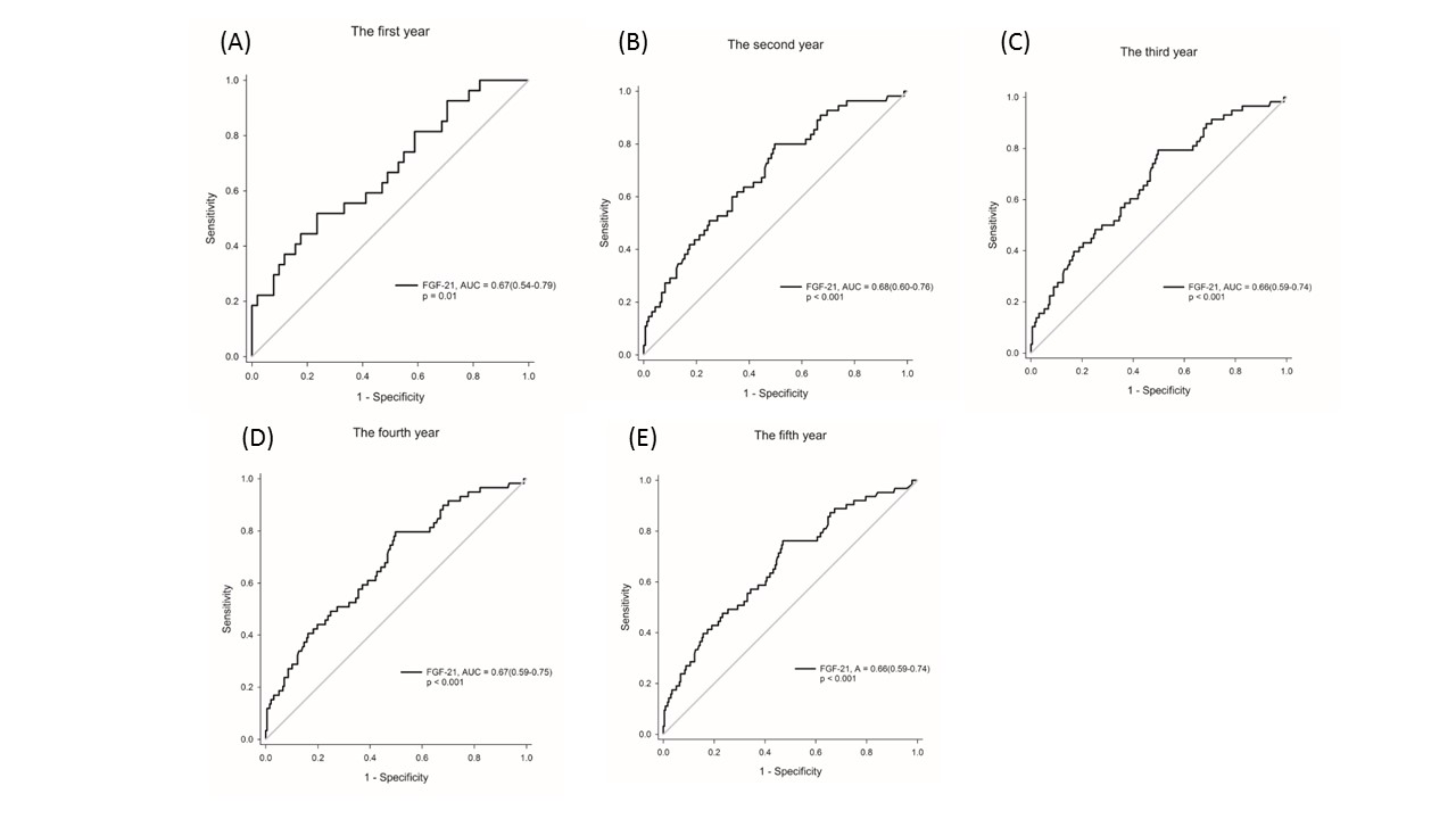

Supplement: Supplementary Figure 1 — Time-dependent receiver operating characteristic curves for prediction of renal events by FGF-21 levels in patients with type 2 diabetes from the first to fifth year (A–E) of follow-up. [file Image_1.tiff]
